# Metabarcoding Reveals Rich and Diverse Aeromycobiota in Protected Oak Forests

**DOI:** 10.1007/s00248-026-02714-5

**Published:** 2026-03-04

**Authors:** Vaidotas Lygis, Adas Marčiulynas, Teodora Plepytė, Sigitas Šulčius, Audrius Menkis

**Affiliations:** 1 State Scientific Research Institute Nature Research Centre, Akademijos str. 2, Vilnius, LT- 08412 Lithuania; 2 Institute of Forestry, Lithuanian Research Centre for Agriculture and Forestry, Liepų str. 1, Girionys, Kaunas, LT-53101 Lithuania; 3 Department of Forest Mycology and Plant Pathology, Uppsala BioCenter, Swedish University of Agricultural Sciences, P.O. Box 7026, Uppsala, SE-75007 Sweden

**Keywords:** Airborne Fungal Communities, High-Throughput Sequencing, Old-Growth Forest, Passive Spore Traps, Species Richness, Spore Deposition

## Abstract

Old-growth oak (*Quercus robur*) forests in Europe are biodiversity hotspots, yet their airborne fungal diversity remains poorly studied. We investigated aeromycobiota in three Lithuanian oak stands (Punia, Dūkštos and Šilinė) using passive spore traps combined with DNA metabarcoding. Weekly sampling between August and September 2022 yielded 75 spore samples, producing 262,755 high-quality fungal sequences clustered into 1,881 operational taxonomic units (OTUs) representing six phyla and 36 classes. Ascomycota (53.1% of OTUs) and Basidiomycota (44.3%) dominated in richness, while Basidiomycota prevailed in relative sequence abundance (62.8%). Major taxa included *Exobasidiaceae* sp., *Cladosporium* sp., *Melampsora* sp., and *Thelephora terrestris*. Airborne fungal communities exhibited a substantial core assemblage shared among stands, accompanied by stand-associated differences in species richness and relative sequence abundance. The Punia stand showed the highest richness and the greatest proportion of stand-specific OTUs. Temporal variation was detectable but moderate relative to spatial differences among stands. Functional guild analysis revealed dominance of saprotrophs (46.5% of assigned OTUs), followed by pathotrophs (14.5%) and symbiotrophs (13.2%). Overall, our results demonstrate that passive spore traps-based airborne metabarcoding captures high fungal diversity and resolves both widespread and stand-associated community patterns in old-growth oak forests. This study provides the first characterization of aeromycobiota in old-growth *Q. robur* forests of Northern Europe and highlights the value of airborne metabarcoding for fungal biodiversity assessment and long-term forest monitoring.

**Supplementary Information:**

The online version contains supplementary material available at 10.1007/s00248-026-02714-5.

## Introduction

Oak-dominated forests are among Europe’s most biodiversity-rich ecosystems [1, 2], yet their extent has declined sharply due to climate change and intensive land use [3]. Old-growth oak stands now persist mainly as small, fragmented remnants [4, 5], which support numerous endangered and specialist organisms because large, old trees provide cavities, deadwood, and other microhabitats essential for their persistence [6, 7].

In Lithuania, oak (*Quercus robur*) stands currently cover 54.6 thousand hectares, or 2.5% of the national forest area, with only ~ 21% under protection [8]. Depending on protection status, felling is either completely prohibited (forest reserves) or restricted, with higher minimum felling ages (140 years in special-purpose forests). Despite slight increases in oak cover in recent decades [8], their condition has been negatively affected by prolonged droughts, lowered groundwater, windstorms, intensive forestry, and urbanisation. As a result, the number of old oaks has declined, placing oak-dependent biota under increasing pressure. Given that pedunculate oak can live for 500–600 years or longer [9] and that many fungal microhabitats develop only after ~ 200 years of age [10, 11], current clear-felling practices at ~ 120 years in non-protected stands largely preclude the establishment of mature oak-associated fungal communities. Oaks support a remarkable range of organisms. More than 330 species are obligately associated with *Q. robur* and its close relative *Q. petraea*, including at least 57 fungal species [12]. In Fennoscandia (Finland, Sweden, and Norway), 253 fungal species are red-listed, about 50 of which are oak-associated [13]. In Lithuania, 117 fungi are included in the national Red Data Book, with 35 strictly linked to *Q. robur* [14]. Many of these species are dependent on old oaks and would face extinction if suitable habitats were lost (e.g) [15, 16]. Continued protection and restoration of oak forests are therefore essential, together with improved understanding of the ecological processes that sustain their biodiversity. Monitoring rare and declining oak-associated species, as well as invasive and quarantine fungi that may threaten forest health, is also of key importance.

Mycological research in European oak forests has increased in recent decades. Many studies have relied on fruitbody surveys (e.g [15, 17–21]), or analysis of oak-associated ectomycorrhizal communities by root-tip morphotyping and molecular identification [21–24]. Other approaches have involved culturing endophytes and pathogens from oak tissues [25–30]. More recently, DNA metabarcoding has transformed microbial biodiversity studies [31]. Environmental DNA (eDNA) methods enable rapid and comprehensive surveys, overcoming limitations of traditional approaches, and allow detection of cryptic or rare species [32–34]. Despite these advantages, eDNA-based studies of fungal communities face several challenges, including false negatives for low-abundance species, limited quantitative interpretability since DNA concentration does not directly reflect organismal abundance, and environmental influences on DNA degradation. Additional complications arise from low template concentrations, high temporal and spatial variability, uncertain source attribution due to long-distance dispersal (particularly in aerobiological studies), increased risk of contamination, and difficulties linking sequence data to formally described species [35, 36]. In oak stands, metabarcoding has been applied to fungi inhabiting oak wood [11, 37], oak stumps [38], oak roots and rhizosphere [39, 40], dead logs [41], bark [42], and experimental wood discs [43]. Few studies, however, have investigated airborne fungal diversity in oak forests, with one example being a study by Chandelier et al. [44] in Belgian stands dominated by *Q. petraea* and *Q. robur*.

Metabarcoding of airborne fungi was pioneered by Fröhlich-Nowoisky et al. [45], who showed that airborne fungal diversity rivals that of soil or plant-associated communities. Subsequent work demonstrated that spore traps capture a broad spectrum of taxa across space and time and provide insights beyond fruitbody surveys or culturing [46]. Airborne mycobiome studies can reveal spatial and temporal drivers of fungal distributions (e.g [47–50]), and have practical applications in disease epidemiology and forecasting [51]. To date, large number of aeromycobiome studies have focused on specific fungal taxa (e.g [52, 53]), human health risks (e.g [54]), or general biodiversity assessments (e.g [50, 55]).

Recent studies have increasingly applied metabarcoding to forest aeromycobiomes across diverse spatial and ecological contexts. These investigations have revealed pronounced spatial, vertical, and seasonal structuring of airborne fungal communities, influenced by forest type, vegetation, and meteorological conditions across temperate and tropical systems in Europe, the Amazon, Japan, and North America [44, 48, 49, 56–62]. Despite these advances, metabarcoding surveys in forest ecosystems remain relatively scarce, and studies targeting the aeromycobiota of biodiversity-rich old-growth forests are particularly limited.

Recent observations suggest that the loss of fungal biodiversity can be as great as in other better-studied groups of organisms [63]. Because fungi are key bioindicators and regulators of ecosystem processes, monitoring their diversity, particularly in oak forests, can provide insights into ecosystem status, resilience, and responses to climate change [63, 64]. This includes the conservation of threatened fungi, which form an essential part of overall biodiversity [65]. Fungal communities are influenced by climate and local meteorological conditions [66]. Temperature, moisture, and substrate availability strongly affect fruitbody production, spore release, and dispersal [62, 67, 68]. As a result, airborne fungal communities often show strong temporal dynamics with marked shifts across days to weeks [48, 58, 69].

In this study, we investigated airborne fungal communities in three protected old-growth *Q. robur* stands in Lithuania using passive spore traps and DNA metabarcoding. We specifically asked: (i) how diverse are airborne fungal communities in old-growth oak forests, and (ii) how fungal community richness and composition vary among spatially separated stands and across short temporal gradients. We hypothesized that: (1) oak stands would share a core set of airborne fungal taxa, but local stand characteristics would influence taxonomic richness and quantitative composition of the fungal communities; (2) temporal variation in fungal communities would be pronounced, reflecting sporulation dynamics; and (3) saprotrophs would dominate the airborne fungal communities with pathogens and symbionts comprising smaller yet consistently detectable components of the assemblage. By combining passive spore trapping with high-throughput sequencing, this study provides the first detailed insight into the aeromycobiota of North European old-growth oak forests, offering new perspectives for biodiversity monitoring, conservation, and ecosystem management.

## Methods

### Study Sites 

The study was conducted in three oak stands in Lithuania: Punia (Alytus district, N 54°30′50.1″, E 24°04′51.6″), Dūkštos (Vilnius district, N 54°49′57.2″, E 24°57′15.0″), and Šilinė (Jurbarkas district, N 55°5′22.58″, E 22°57′13.85″). All sites are located within protected zones (forest reserves or special-purpose forest stands) of local nature conservation areas. The stands were dominated by old (> 200 years) *Q. robur* trees and are known to host several fungal species listed in the Lithuanian Red Data Book [14, 70]. The stands at Punia and Dūkštos exhibited similar tree species composition, being dominated by *Q. robur* with a minor admixture of *Picea abies* and *Alnus incana*, and an understory composed primarily of *Corylus avellana*, *Sorbus aucuparia*, and *Ulmus* spp. The ground vegetation was characterized by *Aegopodium podagraria*, *Urtica dioica*, and various *Carex* species. In contrast, the Šilinė site represented a pure *Q. robur* stand, with only occasional *Prunus padus* individuals in the understory and a herbaceous layer dominated by *Carex* spp. The Punia and Šilinė stands occur on fertile, well-drained soils with normal moisture conditions. The Dūkštos stand is likewise on normally humid soils but of markedly higher fertility than the other two sites. The distances between the study sites were approximately 70 km (Punia–Dūkštos), 100 km (Punia–Šilinė), and 130 km (Dūkštos–Šilinė).

Each trap consisted of a sterile 9-cm Munktell filter paper disc (cotton fibers, particle retention 5–6 μm, class 1 F; Ahlstrom-Munksjö, Stockholm, Sweden) placed between two 10 × 10 cm stainless-steel grates (mesh size 1 × 1 cm) and mounted horizontally on a wooden stake to secure the trap in place. The height of each filter was adjusted to 0.8 m above ground level. It should be noted that passive spore traps, such as used in the present study, function as general collectors of airborne biological particles. In addition to spores, they may also capture other fungal propagules such as hyphal fragments or lichen vegetative propagules, as well as miscellaneous airborne eDNA [36, 47]. In each stand, spore traps were placed at five designated sampling points along a 300-m transect (see [53] for details). The traps were installed on 23 August 2022, and filters were replaced weekly for five consecutive weeks (30 August, 7 September, 13 September, 21 September, and 27 September 2022). In total, 75 samples were obtained (25 per stand). Air temperature and relative humidity were continuously recorded at each site using Uni-T UT330C data loggers (Uni-Trend Technology, Dongguan, China).

### DNA extraction, PCR amplification and sequencing

To avoid contamination, all spore traps and filter paper discs were handled exclusively with sterile disposable gloves during field installation, collection, and laboratory processing. Filter discs were supplied sterile and were opened, transferred, and prepared for DNA extraction under sterile conditions in a dedicated microbiology laboratory. All laboratory procedures, including unpacking of collected samples (filter paper discs), DNA extraction, and PCR setup, were carried out under laminar-flow hoods to minimize the risk of contamination. In the laboratory, the collected samples were freeze-dried, homogenized, and total DNA was extracted using a CTAB protocol [49]. The extracted DNA was cleaned using NucleoSpin^®^Soil kit (Macherey-Nagel GmbH & Co. Duren, Germany). The ITS2 rRNA region was amplified using the fungal-specific primer gITS7 [71] and the universal primer ITS4 [72], both with integrated barcodes for sample identification. The barcodes were 8 bp long and designed to differ by at least 2 bases from one another to minimize the risk of misassignment due to sequencing errors. PCR was performed on Applied Biosystems 2720 thermal cycler (Applied Biosystems, Foster City, CA, USA) with DreamTaq Green polymerase (Thermo Scientific, Waltham, MA, USA) under standard cycling conditions (95 °C 5 min; 27 cycles of 95 °C 30 s, 56 °C 30 s, 72 °C 30 s; final extension 72 °C 7 min). PCR products were purified using sodium acetate/ethanol precipitation and quantified with a Qubit 4.0 fluorometer (Life Technologies). During PCR amplification, a negative control (milliQ water) was included to monitor potential contamination on electrophoresis gels. Only PCR runs that consistently yielded no product in negative controls (which typically showed no product) were included for sequencing. Amplified products were pooled in equimolar concentrations and sequenced using two SMRT cells on the PacBio Sequel platform at SciLifeLab (Uppsala, Sweden).

### Bioinformatics

Raw ITS2 sequences were processed using the SCATA NGS pipeline (https://scata.mykopat.slu.se). Quality filtering excluded sequences shorter than 200 bp, low- or medium-quality reads, primer dimers, and sequences with long homopolymers (> 3 bp, trimmed prior to clustering). The > 3 bp homopolymer cutoff was applied because PacBio CCS reads are prone to insertion/deletion errors in homopolymeric regions, even in relatively short amplicons such as ITS2 (e.g [73–75]). Low- and medium-quality reads were defined as sequences with a substantial proportion of bases below *phred* quality score 20 (Q < 20 [76]), including those containing excessive ambiguous nucleotides (N). Reads missing barcodes or primers were also discarded. To reduce noise from low-frequency artefacts, singleton reads were removed prior to diversity analyses. The remaining sequences were clustered into operational taxonomic units (OTUs) using single-linkage clustering with a ≥ 99% similarity threshold, and only OTUs represented by two or more high-quality sequences across the dataset were retained. Rarefaction curves were generated from the resulting dataset to evaluate sampling completeness and to assess whether sequencing depth was sufficient to capture a substantial proportion of fungal diversity (Fig. S1). Rarefaction was used diagnostically to assess sampling adequacy rather than as a normalization procedure; diversity indices were calculated from SRS-normalized data to account for differences in sequencing depth.

Taxonomic assignment was carried out manually using BLASTn searches against the GenBank (NCBI) and UNITE [77] databases. The following thresholds were applied: ≥80% sequence query coverage and ≥ 50% similarity for phylum-level identification; ≥70% for class; ≥81% for order; ≥88% for family; ≥97% for genus; and ≥ 99% for species. These thresholds follow empirical ITS similarity ranges proposed and applied in previous studies (e.g [78, 79]), and reflect commonly used values in fungal metabarcoding, with more stringent cutoffs applied at lower taxonomic ranks (e.g [79–81]). Non-fungal OTUs were removed after taxonomic assignment by excluding all sequences whose highest-scoring BLASTn matches in GenBank corresponded to non-fungal lineages (*Plantae*, *Chromista*, *Amoebozoa*, *Protozoa*) and by confirming the absence of plausible fungal affiliations in UNITE. OTUs lacking fungal hits among the top BLASTn alignments were also discarded. Taxonomic identities of retained OTUs were verified manually using NCBI and UNITE taxonomy. Fungal OTUs with < 81% similarity to reference sequences were considered insufficiently resolved for order-level identification and were retained as unclassified fungal lineages (‘Unidentified sp.’) with unique identifiers. Taxon names were updated manually according to MycoBank (www.mycobank.org).

Fungal taxa were further assigned to trophic groups and functional guilds using the FUNGuild database [82], followed by manual curation from the literature. Trophic modes were defined as pathotrophic, saprotrophic, symbiotrophic, or combinations thereof, resulting in seven distinct trophic categories. Sequences assigned to the class *Malasseziomycetes* were excluded, as these fungi are obligate animal-associated taxa (skin-infecting fungi) and likely contaminants [83].

### Statistical Analysis

All analyses were implemented in R (version 4.5.2) using RStudio (version 2025.09.2 + 418; RStudio PBC, Boston, USA), with analysis code provided in the *Supplementary Materials* (Supplementary_File_3_Rscripts.zip). Qualitative Sørensen similarity indices were calculated separately in Microsoft Excel from presence–absence OTU data using the standard Sørensen-Dice formula. OTU read counts were treated as proxies for relative sequence abundance rather than absolute propagule abundance. While sequence counts are not strictly quantitative and may be influenced by methodological and biological factors, relative abundances can capture ecologically meaningful patterns for community comparisons [59, 84], provided they are interpreted cautiously [55, 85]. Differences in temperature and humidity between forest stands were evaluated using Welch’s ANOVA with Games-Howell post-hoc tests after assessment of data distribution and homogeneity of variances.

### Alpha Diversity

Alpha diversity was assessed using three complementary metrics: observed species richness, Shannon entropy (*H’*), and Simpson’s index (1-D). Prior to analysis, data were normalized to sequencing depth of 350 reads per sample (retaining ca. 88% of samples) using SRS procedure (scaling with ranked-subsampling; [86]) to balance diversity representation across forest stands. Rarefaction analysis was performed using rarefaction curves available from *vegan* package v2.7-2 and sample-size-based extrapolation with the *iNEXT* package v3.0.2 [87]. The iNEXT analysis was conducted across three Hill number orders (q = 0, 1, 2) with 100 bootstrap replicates and 95% confidence intervals. Statistical differences in alpha diversity between forest stands were evaluated using mixed-effects models implemented in the *betta_random* function from the *breakaway* package v4.8.4 [88] using stand as a fixed effect and Dūkštos stand as the reference level, while the sampling period was included as a random effect to account for temporal variation. For species richness, estimates and standard errors were obtained using Chao1-based approximations. Standard errors for Shannon and Simpson indices were estimated via bootstrap resampling (500 iterations). Multiple comparisons were corrected using the Benjamini-Hochberg false discovery rate (FDR) procedure.

### Beta Diversity

Differences in community composition were tested using permutational multivariate analysis of variance (PERMANOVA) with Aitchison distance matrix (Euclidean distance after centered log-ratio transformation (rCLR; [89]) using the *adonis2* function with 9,999 permutations in *vegan* package v2.7-2. The forest stand, sampling period, and stand and period interaction were used as predictors. Post-hoc pairwise comparisons were conducted using the *pairwise.adonis2* function from the *pairwiseAdonis* package v0.4 with 9,999 permutations to examine the effect of sampling period within each forest stand. The similarity percentage procedure (SIMPER) implemented in *vegan* package (v2.7-2.) was used to analyze the contribution of taxa to the mean dissimilarity percentage between forest stands. Community composition data was visualized using non-metric multidimensional scaling (NMDS) with the *metaMDS* function in *vegan* package v2.7-2. NMDS was performed in two dimensions (k = 2) with up to 1,000 random starts to ensure convergence. The homogeneity of dispersions was tested using the *betadisper* function [90]. Distances from samples to their group centroid were calculated for forest stand, sampling period, and their interaction. Statistical significance of dispersion differences was assessed using permutation tests (9,999 permutations). Differential abundance analysis was done with *MaAsLin3* package v1.2.0 [91] using model ~ STAND + (1|PERIOD) with Dūkštos forest stand as the reference level, and OTU thresholds for abundance of 0.0001 and prevalence of 0.05 across samples. Significance was determined using Benjamini-Hochberg FDR correction with a threshold of q < 0.25, following the recommendations for exploratory microbiome analyses. Both individual model q-values (testing abundance or prevalence separately) and joint q-values (testing whether either abundance or prevalence differs) were reported.

## Results

A total of ca. 1.9 million DNA sequences were generated by PacBio sequencing. After initial quality filtering, 0.89 million reads (47.0%) met our high-quality criteria (*phred* ≥ Q20) and were retained, whereas 53.0% of reads were discarded as low-quality. Subsequent filtering and clustering further refined the dataset: removal of singleton reads (10,855 reads; 1.2% of all high-quality reads), exclusion of non-fungal and unassigned OTUs (376,458 reads; 42.2%), and exclusion of potential fungal contaminants assigned to *Malasseziomycetes* (243,220 reads; 27.2%). Additional sequencing and taxonomic assignment statistics are provided in Table S1. After all processing steps, the final dataset comprised 262,755 high-quality fungal reads (29.4%) with an average read length of 277 bp (range 207–552 bp), representing 1,881 OTUs (Table S2). Rarefaction and extrapolation curves showed diminishing returns in fungal taxon accumulation (q = 0) with increasing sequencing depth, without reaching a clear asymptote, indicating substantial but incomplete recovery of rare taxa (Fig. S1a). In contrast, Shannon (q = 1) and Simpson (q = 2) diversity rapidly stabilized across stands (Fig. S1b, c), suggesting that diversity patterns driven by common and abundant taxa were well captured and relatively insensitive to sequencing depth.

**Fig. 1:**
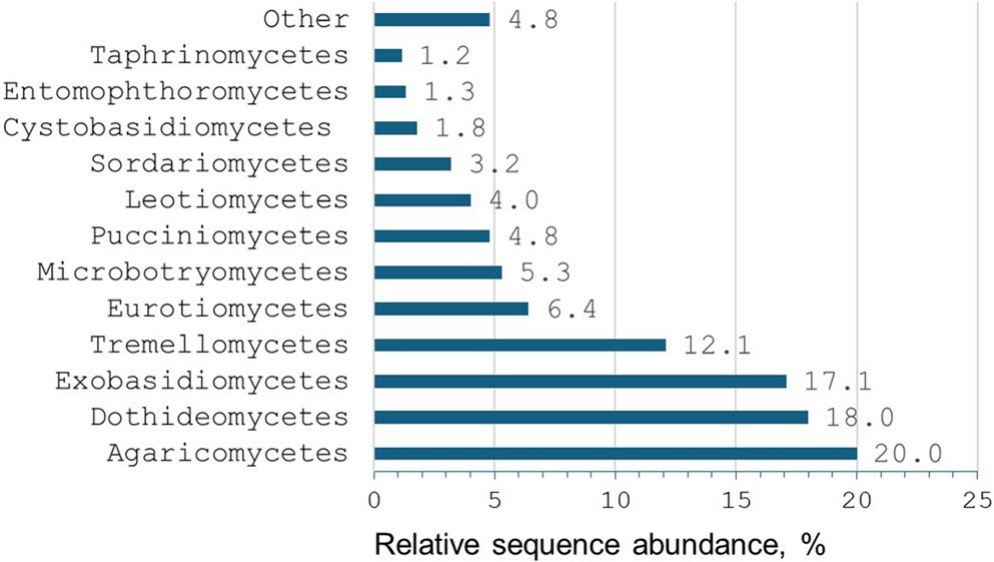
Relative sequence abundance (%) of fungal taxonomic classes in airborne samples from old-growth oak stands (combined data from Šilinė, Punia, and Dūkštos). Values are based on high-quality fungal DNA reads; “Other” denotes classes with < 1% relative abundance

All 1,881 OTUs were assigned to six fungal phyla (Fig. S2), dominated by Ascomycota (53.1%) and Basidiomycota (44.3%), with 98.9% classified into 36 classes. Of the 1,881 OTUs, 605 (32.2%) could be identified to species level, 374 (19.9%) to genus level, 424 (22.5%) to family level, and 314 (16.7%) to order level. The remaining 165 OTUs (8.8%) were named as ‘Unidentified sp.‘, given a unique identification number and assigned to the appropriate taxonomic phylum or class (if known) (Table S2). The richest classes were *Agaricomycetes*, *Dothideomycetes*, *Sordariomycetes*, and *Leotiomycetes*, with *Agaricomycetes* also dominating in relative sequence abundance (20.0%), followed by *Dothideomycetes* (18.0%), *Exobasidiomycetes* (17.1%), and *Tremellomycetes* (12.1%) (Fig. 1, Tables S4-S6).

### Stand-level Patterns of Fungal Alpha Diversity

**Fig. 2:**
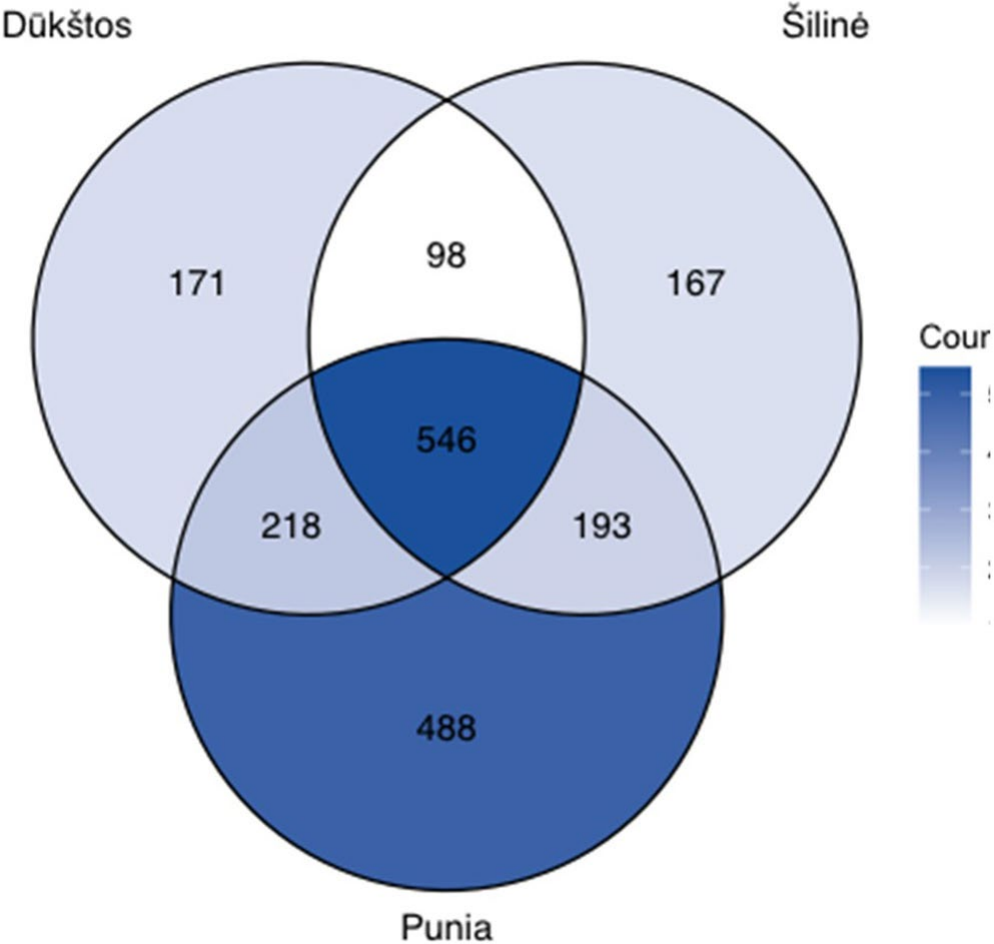
A Venn diagram showing the fungal taxonomic diversity (OTU number) and overlap among the studied oak stands of Šilinė, Punia and Dūkštos

Rarefaction analysis indicated clear differences in fungal species richness among the three oak stands (Fig. S1a). Punia exhibited the highest richness, with 1,445 observed OTUs and an estimated richness of 1,512 OTUs derived from iNEXT extrapolated rarefaction curves (95% confidence interval [CI]: 1,473–1,552). This was followed by Dūkštos (1,033 observed OTUs; estimated 1,160 OTUs; 95% CI: 1,114–1,206) and Šilinė (1,004 observed OTUs; estimated 1,092 OTUs; 95% CI: 1,048–1,135). The iNEXT analysis results are provided in Supplementary File 2. The higher richness observed in Punia coincided with a larger proportion of unique taxa (25.9% of all OTUs), compared with Dūkštos (9.1%) and Šilinė (8.9%) (Fig. 2).

**Fig. 3:**
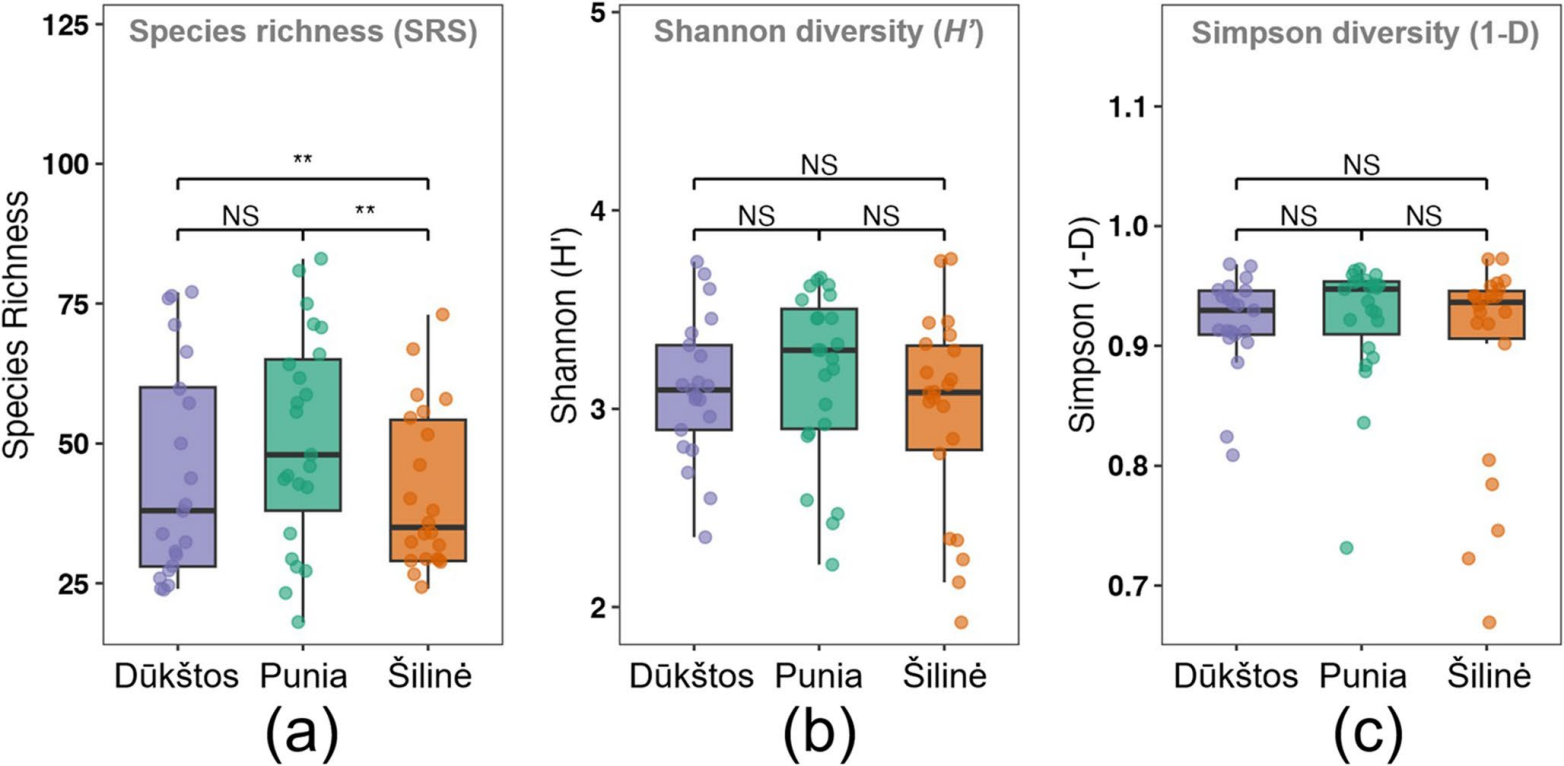
Alpha diversity of airborne fungal communities across the studied oak stands of Dūkštos, Punia and Šilinė. (**a**) species richness, (**b**) Shannon diversity (H′), and (**c**) Simpson diversity (1−D) following SRS normalization to 350 reads per sample. Differences among stands were assessed using linear mixed-effects models with sampling period as a random effect. Boxes show medians and interquartile ranges; points represent individual samples. Significance of pairwise comparisons is indicated above plots (**q < 0.01; NS, not significant; Benjamini–Hochberg corrected)

Rarefaction and extrapolation of Shannon (q = 1) and Simpson (q = 2) diversity indicated that community patterns dominated by common and abundant taxa were well captured in all three oak stands, with curves rapidly approaching asymptotes and showing broadly similar trajectories (Fig. S1b, c). This suggests that differences among stands were more pronounced for rare taxa than for common or dominant components of the airborne fungal communities. Alpha diversity indices calculated from SRS-normalized data were broadly consistent with the rarefaction results (Fig. 3). Species richness tended to be higher in Punia, with significant pairwise differences detected between Punia and Šilinė, as well as between Šilinė and Dūkštos (q < 0.01; Fig. 3a). In contrast, Shannon and Simpson diversity indices showed substantial overlap among stands and no clear or consistent differences (Fig. 3b, c), suggesting similar within-stand community complexity and dominance structure.

Environmental conditions differed modestly among stands during the sampling period. Punia exhibited significantly higher mean relative humidity (87.8 ± 0.4%) compared with Dūkštos (85.6 ± 0.4%) and Šilinė (80.0 ± 0.5%) (Welch ANOVA: F = 74.27, *p* < 0.001), whereas mean air temperature did not differ significantly among sites (range: 13.0–13.6 °C; Welch ANOVA: F = 2.56, *p* = 0.078) (Table S3; Fig. S3).

Overall, alpha-diversity patterns indicated limited differentiation among stands beyond species richness. While Punia consistently tended to exhibit higher richness, Shannon and Simpson diversity showed broadly overlapping distributions across all stands, indicating comparable evenness and dominance patterns. Because all diversity metrics were calculated from SRS-normalized data, these patterns are unlikely to be driven by variation in sequencing depth.

### Community Variation among Oak Stands

Across all three stands, *Agaricomycetes*, *Dothideomycetes*, *Sordariomycetes*, *Leotiomycetes*, and *Eurotiomycetes* contained the highest numbers of OTUs when taxonomic richness was summarized by fungal class (Fig. S4a). In contrast, relative sequence abundance patterns varied more strongly among stands (Fig. S4b). In particular, Punia showed elevated contributions of *Exobasidiomycetes*, *Tremellomycetes*, and *Microbotryomycetes* relative to the other sites.

**Fig. 4:**
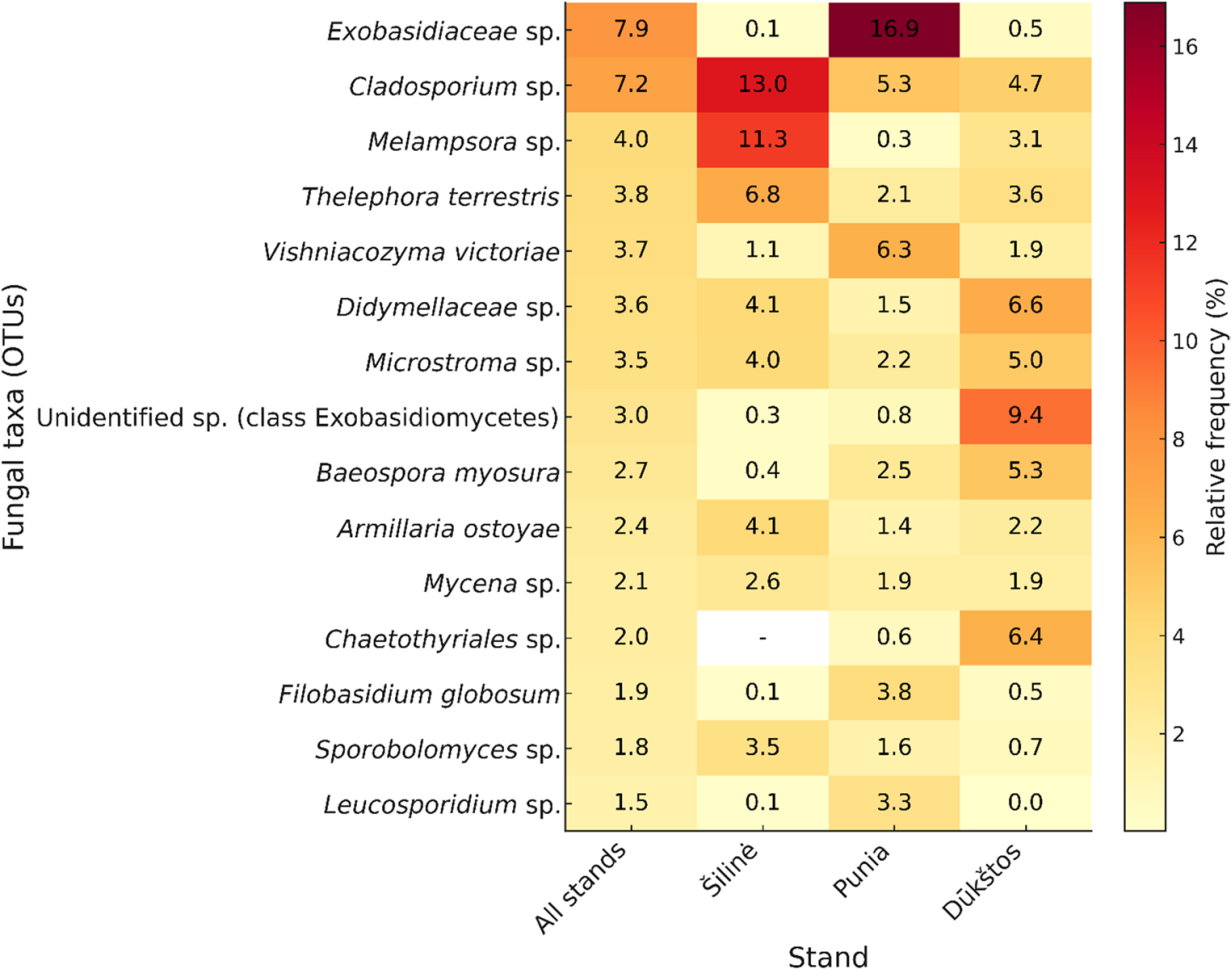
Heatmap showing the relative abundances (%) of the 15 most abundant fungal OTUs across three old-growth oak stands in Lithuania (Šilinė, Punia, and Dūkštos). Warmer colors indicate higher relative sequence abundances. Distinct dominance patterns are: *Exobasidiaceae* sp. and *Vishniacozyma victoriae* in Punia, *Cladosporium* sp. and *Melampsora* sp. in Šilinė, and Unidentified sp. together with *Didymellaceae* sp. and *Chaetothyriales* sp. in Dūkštos

Analysis of the 15 most abundant OTUs revealed clear differences in dominance patterns among stands (Fig. 4). In Punia, *Exobasidiaceae* sp. and *Vishniacozyma victoriae* were most abundant, together accounting for 22.2% of all reads. Šilinė was characterized by high relative sequence abundances of *Cladosporium* sp. and *Melampsora* sp. (24.3% combined), with *Thelephora terrestris* also contributing substantially. In contrast, Dūkštos was dominated by an unidentified taxon within *Exobasidiomycetes*, *Didymellaceae* sp., and *Chaetothyriales* sp., each contributing more than 6–9% of reads, while *Baeospora myosura* and *Microstroma* sp. were also common. Several taxa, including *Thelephora terrestris*, *Vishniacozyma victoriae*, *Didymellaceae* sp., *Microstroma* sp., *Armillaria ostoyae*, *Baeospora myosura*, and *Mycena* sp., were consistently detected across all stands, although their relative sequence abundances varied.

**Fig. 5:**
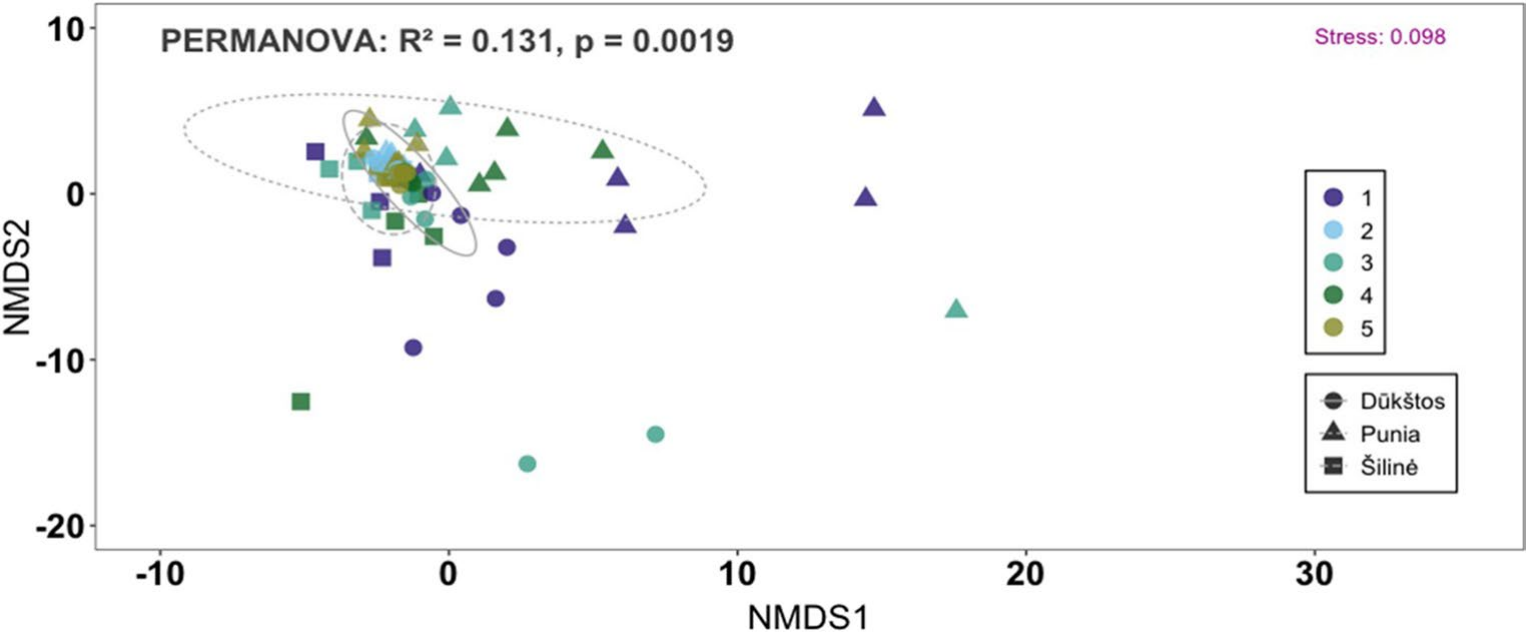
Non-metric multidimensional scaling (NMDS) ordination of airborne fungal community composition in the studied oak stands of Šilinė, Punia and Dūkštos based on Aitchison distance (Euclidean distance on rCLR-transformed data). Samples are colored by sampling period (1–5; see Methods for sampling dates) and shaped by oak stand; ellipses represent 95% confidence intervals around stand centroids. PERMANOVA results and stress value are shown on the plot

Multivariate analyses indicated structuring of airborne fungal community composition across samples, accompanied by substantial overlap among oak stands (Fig. 5). NMDS ordination showed broadly overlapping communities from Punia, Šilinė, and Dūkštos, with only weak stand-related separation. The two-dimensional ordination provided a good representation of the data (stress = 0.098). PERMANOVA revealed that temporal variation explained a significant proportion of community compositional variation (R² = 0.131, *p* = 0.0019; Fig. 5), whereas stand-level differences were comparatively weaker after accounting for sampling period effects (see Supplementary File 2).

When forest stand and sampling period effects were evaluated jointly, PERMANOVA indicated statistically significant effects of stand (R² = 0.06, pseudo-F = 2.74, *p* < 0.001), sampling period (R² = 0.11, pseudo-F = 2.49, *p* < 0.001), and their interaction (R² = 0.13, pseudo-F = 1.42, *p* = 0.002), although effect sizes were moderate (see Supplementary File 2). NMDS patterns and pairwise comparisons further indicated that fungal communities in Punia exhibited relatively higher variability across both spatial and temporal scales compared with Šilinė and Dūkštos.

**Fig. 6:**
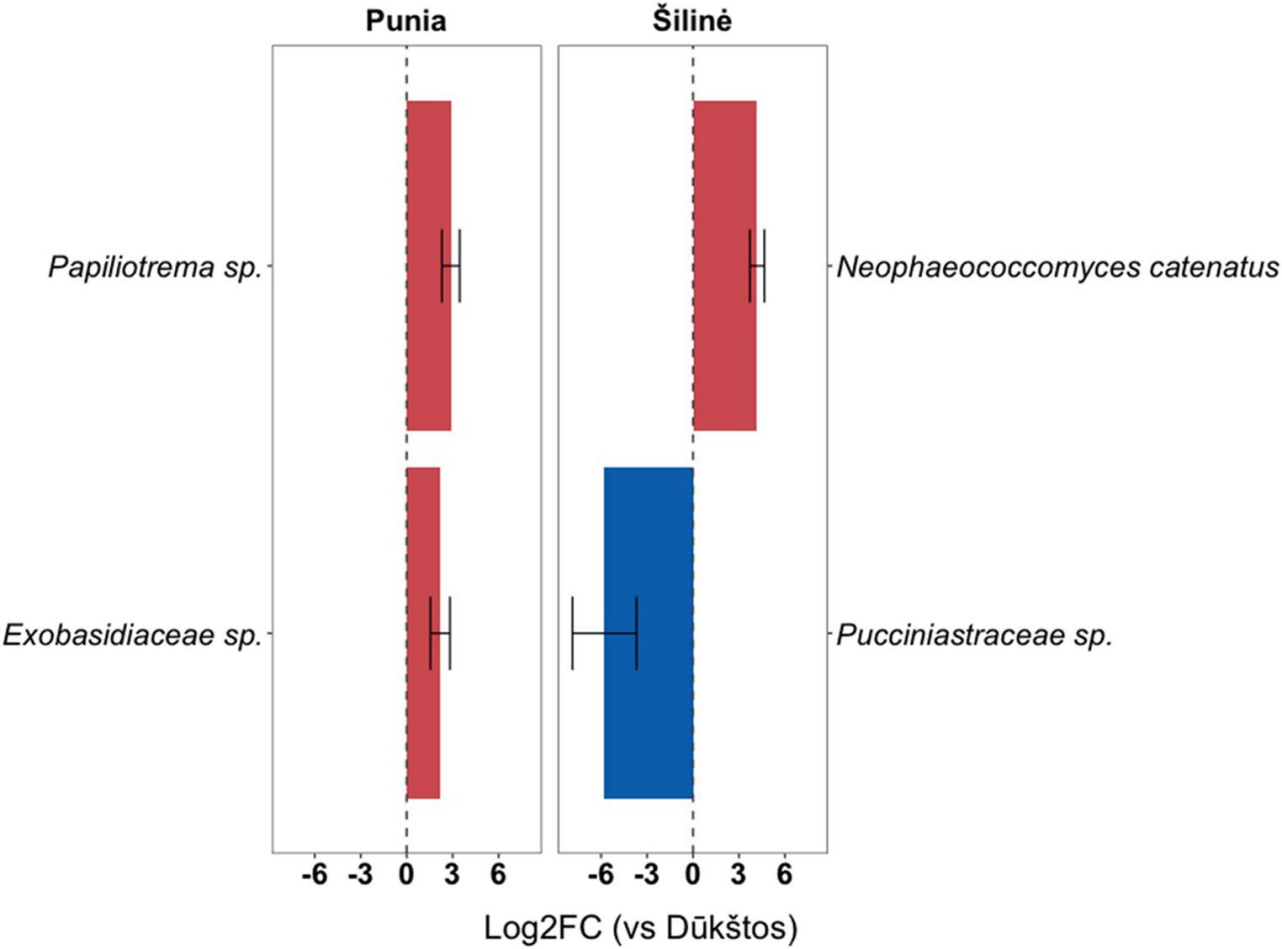
Differential abundance analysis identifying airborne fungal taxa enriched or depleted in Punia and Šilinė oak stands relative to the reference stand Dūkštos, expressed as log2 fold changes with confidence intervals

Differential abundance analysis identified several taxa showing stand-associated shifts in relative sequence abundance (Fig. 6). Relative to the reference stand Dūkštos, Punia was enriched in *Papiliotrema* sp. and *Exobasidiaceae* sp., whereas Šilinė was enriched in *Neophaeococcomyces catenatus* and depleted in *Pucciniastraceae* sp. The relative occurrence of these differentially abundant taxa varied across sampling periods, as illustrated by temporal heatmap patterns (Fig. S5). In addition, the SIMPER analysis, which calculates the percentage contribution of each taxon to the overall dissimilarity between study sites, revealed that *Thelephora terrestris*, *Exobasidiaceae* sp., *Baeospora myosura*, *Melampsora* sp., *Cladosporium* sp. were the top 5 taxa that contributed the most to the observed differences between the investigated stands (see Supplementary File 2).

Qualitative Sørensen similarity indices indicated relatively high species overlap among stands (0.60–0.63), consistent with the shared core assemblage visualized in the Venn diagram (Fig. 2). Overall, while Punia tended to exhibit higher OTU richness and relative sequence abundance (Table 1; Fig. S1), differences in airborne fungal community composition among stands were generally moderate and context-dependent, reflecting taxon-specific shifts rather than wholesale community turnover.

**Table 1:** Numbers and relative abundances of high-quality fungal DNA sequence reads, and numbers of determined operational taxonomic units (OTUs) categorized by fungal phyla in the investigated oak stands of Šilinė, Punia and Dūkštos, based on samples collected by passive spore traps during August-September 2022

Phylum	Šilinė			Punia			Dūkštos			All stands		
	No. of sequence reads^a^	Relative sequence abundance^b^ (%)	No. of OTUs	No. of sequence reads	Relative sequence abundance (%)	No. of OTUs	No. of sequence reads	Relative sequence abundance (%)	No. of OTUs	No. of sequence reads	Relative sequence abundance (%)	No. of OTUs
*Ascomycota*	29,906	42.38	592	34,107	28.44	748	27,846	38.54	590	91,859	34.96	999
*Basidiomycota*	39,330	55.73	385	82,337	68.65	662	43,457	60.15	413	165,124	62.84	833
*Chytridiomycota*	18	0.03	2	22	0.02	4	31	0.04	4	71	0.03	7
*Mucoromycota*	595	0.84	20	423	0.35	23	792	1.10	21	1,810	0.69	32
*Olpidiomycota*	83	0.12	1	41	0.03	1	28	0.04	1	152	0.06	1
*Zoopagomycota*	635	0.90	4	3,009	2.51	7	95	0.13	4	3,739	1.42	9
All phyla	70,567	100	1,004	119,939	100	1,445	72,249	100	1,033	262,755	100	1,881

^a^the number of high-quality ITS2 rRNA sequence reads generated by PacBio sequencing

^b^the proportion of all high-quality fungal DNA sequence reads

### Temporal Variation

**Fig. 7:**
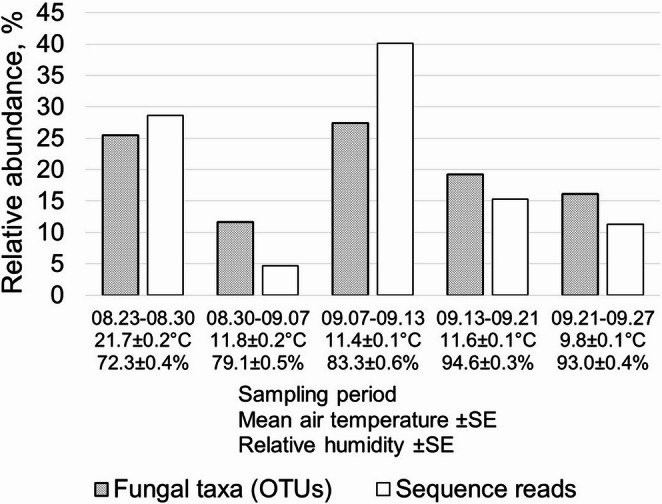
Dynamics of relative abundances of identified fungal taxa (OTUs) and high-quality DNA sequence reads across five sampling periods from August to September 2022 (x axis) in the studied oak stands of Šilinė, Punia and Dūkštos (combined data for all stands). Values for mean air temperature (± standard error) and relative humidity (± standard error) for each sampling period are displayed beneath the x-axis labels

Temporal variation in airborne fungal community composition was evident across sampling periods but lacked a consistent or directional pattern. NMDS ordination showed substantial overlap among periods, with no clear discrete temporal clustering in ordination space (Fig. 5). Marked temporal fluctuations were observed in both OTU richness and relative sequence read counts (Fig. 7; Table 2). A pronounced decline in taxonomic richness and read counts occurred between the first (23–30 August) and second (30 August–7 September) sampling periods, followed by a sharp increase during the subsequent period (7–13 September). This increase coincided with lower mean air temperatures and higher relative humidity. The highest OTU richness and sequence read counts were recorded during 7–13 September (1,256 OTUs), whereas the lowest values were observed during 30 August–7 September (537 OTUs).

**Table 2:** Numbers and relative abundances of high-quality fungal DNA sequence reads, and numbers of determined operational taxonomic units (OTUs) in the investigated oak stands of Šilinė, Punia and Dūkštos, based on samples collected by passive spore traps during August-September 2022

Sampling period (dates)	Šilinė				Punia				Dūkštos				All stands			
	No. of sequence reads^a^	Relative sequence abundances^b^ (%)	No. of OTUs		No. of sequence reads	Relative sequence abundances (%)	No. of OTUs		No. of sequence reads	Relative sequence abundances (%)	No. of OTUs		No. of sequence reads	Relative sequence abundances (%)	No. of OTUs	
08.23–08.30	12,862	18.23	475		38,488	32.09	814		23,830	32.98	620		75,180	28.61	1,172	
08.30–09.07	3,749	5.31	315		3,421	2.85	332		5,225	7.23	269		12,395	4.72	537	
09.07–09.13	15,286	21.66	392		51,595	43.02	919		38,544	53.35	658		105,425	40.12	1,256	
09.13–09.21	19,734	27.96	523		18,196	15.17	608		2,176	3.01	314		40,106	15.26	885	
09.21–09.27	18,936	26.83	499		8,239	6.87	364		2,474	3.42	295		29,649	11.28	738	
All periods	70,567	100	1,004		119,939	100	1,445		72,249	100	1,033		262,755	100	1,881	

^a^the number of high-quality ITS2 rRNA sequence reads generated by PacBio sequencing

^b^the proportion of all high-quality fungal DNA sequence reads

Temporal shifts were also apparent at the class level (Fig. S6; Table S6). *Agaricomycetes* and *Dothideomycetes* consistently exhibited high taxonomic richness across all sampling periods. In contrast, relative sequence abundance varied more strongly over time, with *Exobasidiomycetes*, *Tremellomycetes*, *Eurotiomycetes*, and *Pucciniomycetes* intermittently dominating the airborne fungal assemblage, particularly during the 7–13 September sampling period.

### Trophic and Functional Groups

**Fig. 8:**
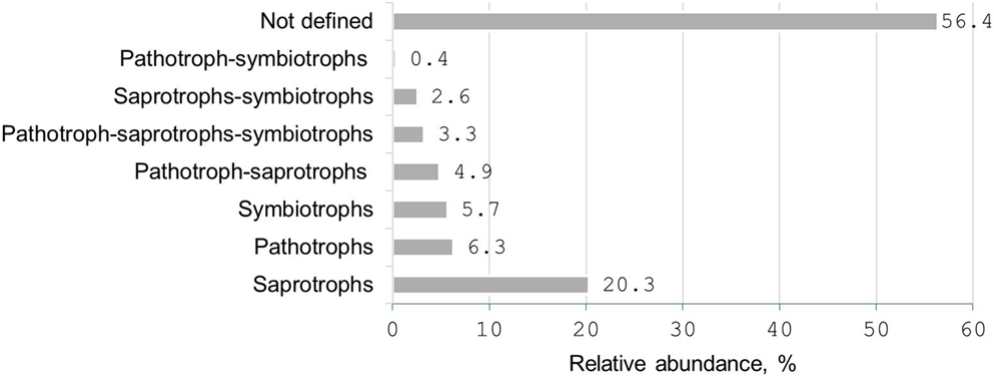
Relative abundance (%) of fungal OTUs assigned to trophic groups in airborne fungal communities captured by passive spore traps in the studied oak stands of Šilinė, Punia and Dūkštos (combined data for all stands)

Of the 1,881 OTUs, 821 (43.6%) could be assigned to trophic groups or functional guilds (Table S4). The rest of the OTUs (56.4%) could not be assigned due to insufficient taxonomic resolution or ecological data. Across all stands, saprotrophs dominated (382 OTUs, 46.5%), followed by pathotrophs (119, 14.5%), symbiotrophs (108, 13.2%), and mixed-mode groups (e.g., pathotroph–saprotrophs, 92 OTUs, 11.2%) (Fig. 8).

Functional assignment revealed 215 OTUs as plant pathogens (26.2% of all classified OTUs), with *Agaricomycetes* contributing the most (49 OTUs), often as weak opportunistic pathogens. In terms of taxonomic richness, the variation in representation of functional groups was not high among the stands: saprotrophs and plant pathogens consistently showed the highest richness peaking in Punia (Fig. S7a). In terms of relative sequence abundance, the variation was similar but more pronounced, with saprotrophs dominating in Punia and Dūkštos, and plant pathogens - in Šilinė (Fig. S7b). 

### Most Common, Red-Listed, Invasive, and Quarantine Species

The most common taxa (in terms of relative sequence abundance) identified by us to species level were an ectomycorrhizal basidiomycete *Thelephora terrestris* (3.8% of all OTUs), a saprotrophic basidiomycetous yeast *Vishniacozyma victoriae* (3.8%), a saprotrophic basidiomycete *Baeospora myosura* (2.7%), an important basidiomycetous tree pathogen *Armillaria ostoyae* (2.4%), all occurring at relatively high yet uneven frequencies across the investigated stands (Fig. 4; Table S2). Apart from *A. ostoyae*, the most common plant-pathogenic species included a maple tar spot-causing ascomycete *Rhytisma acerinum* (1.1%) and wood-decay-causing polypore *Trametes versicolor* (0.8%).

Four species listed in the Lithuanian Red Data Book [14] were detected: *Fistulina hepatica* (found in all three stands), *Xylobolus frustulatus* (Punia only), *Steccherinum bourdotii* (all stands), and *Fomitopsis rosea* (Punia only). Two species invasive to Lithuania were identified: *Hymenoscyphus fraxineus* (ash dieback pathogen) and *Ustilago maydis* (maize smut), both rare in samples (Table S2). No EU quarantine-listed fungi were detected.

## Discussion

We detected species-rich airborne fungal diversity in old-growth oak stands, recording 1,881 OTUs across three spatially separated sites. Although this richness is higher or comparable to values reported in other European forest aeromycobiome studies (e.g [44, 48, 49, 61, 62]), such cross-study comparisons should be interpreted with caution because differences in sequencing platforms, primer sets, and bioinformatic pipelines - including OTU clustering thresholds - can strongly affect the number of OTUs recovered. Our findings also align with recent work by Nordén et al. [92], who showed that *Q. robur* hosts exceptionally rich and distinctive fungal communities in wood, supporting many unique and red-listed taxa. These results reinforce the view that structural complexity, longevity, and evolutionary associations of oaks confer a disproportionately high fungal reservoir role in temperate forests [11, 12].

The high taxonomic yield obtained during the present study validates the sampling effort and shows that passive spore traps, when run over weekly intervals, can capture a broad spectrum of fungal taxa, including both abundant and rare lineages, despite potential limitations such as spore loss by washout during rain events [46, 93]. It is important to note that our dataset and the resulting rarefaction curves (Fig. S1) were generated after removing singleton OTUs, and therefore likely represent a conservative estimate of airborne fungal diversity. Singleton removal can shift species-accumulation curves and underestimate richness because low-frequency features disproportionately influence diversity metrics [94–96]; however, extensive methodological work has shown that singleton OTUs are often enriched in sequencing artefacts and their retention can obscure ecological patterns [97]. Despite this conservative filtering, rarefaction and extrapolation analyses indicate that a substantial proportion of the fungal community was recovered during the present work. Species accumulation curves (q = 0) showed diminishing returns with increasing sequencing depth, suggesting that additional sequencing would primarily detect further rare taxa, whereas Shannon (q = 1) and Simpson (q = 2) diversity rapidly stabilized across stands. Together, these results indicate that the sequencing depth was sufficient to robustly characterize the dominant and common components of airborne fungal communities despite incomplete recovery of the rarest taxa.

Interpretation of abundance-based patterns also requires caution, as sequence read counts represent relative rather than absolute abundances and may be influenced by non-fungal DNA captured in the traps (e.g [36, 47]). Although the relationship between read number and true organism abundance remains debated [55, 85, 98], metabarcoding data are widely regarded as semi-quantitative and generally more informative for ecological comparisons than presence–absence data alone, with relative sequence abundances often capturing ecologically meaningful patterns [59, 84]. In this context, the observed abundance-based differences among stands are best interpreted as relative shifts in community structure rather than direct measures of propagule density.

Although all three oak stands shared a substantial core assemblage (qualitative Sørensen similarity ~ 0.60–0.63), clear stand-associated differences emerged in the relative sequence abundances of individual taxa. The dominant-OTU heatmap (Fig. 4) and differential abundance analysis (Fig. 5; Fig. S5; Supplementary File 2) consistently highlighted distinct abundance signatures among stands. Punia showed elevated relative abundances of an *Exobasidiaceae* taxon and the basidiomycetous yeast *Vishniacozyma victoriae*, whereas Šilinė was characterized by higher contributions of ascomycetous mold *Cladosporium* sp. and the rust pathogen *Melampsora* sp. Dūkštos, in turn, was distinguished by an unidentified *Exobasidiomycetes* taxon, *Didymellaceae* sp., and *Chaetothyriales* sp. Overall, stand-level differentiation was expressed mainly as taxon-specific shifts in relative sequence abundance rather than as large, uniform differences in overall diversity.

A substantial portion of this site-level heterogeneity can be interpreted through the lens of fungal biology. Numerous studies have shown that many fungi exhibit dispersal limitation despite producing large numbers of propagules, and that distinct community signatures can arise over distances of tens to hundreds of meters [59]. As a result, the fungal assemblage arriving at any given location reflects not only local environmental conditions but also the broader biotic and abiotic context of the surrounding landscape. Substrate availability, soil properties, vegetation structure, host distribution, and successional stage all influence which propagules are present and capable of establishing (for references see [59]). In addition, stochastic processes, particularly priority effects, may reinforce differences among nearby patches by conferring persistent advantages to early-arriving taxa [98]. Together, these processes provide a mechanistic explanation for the moderate, taxon-specific spatial differentiation observed among oak stands in this study, whereby airborne communities represent filtered subsets of the regional species pool shaped by dispersal constraints, landscape structure, and contingent assembly dynamics.

The comparatively higher species richness and greater proportion of stand-specific OTUs observed in the Punia stand may partly reflect its status as a Strict Nature Reserve (Punios Šilas), characterized by long-term forest continuity, high relative humidity, and relatively stable microclimatic conditions [70]. Long-standing structural continuity, the presence of large and old oaks, minimal disturbance, and the retention of diverse microhabitats may favor the accumulation and persistence of fungal taxa, including rare or sporadically detected species. In contrast, Dūkštos and Šilinė are substantially smaller forest fragments (36 and 300 ha, respectively, compared to 2,237 ha for Punios Šilas Strict Nature Reserve) and may be more influenced by edge effects or localized disturbance regimes, potentially limiting the long-term persistence of some taxa. Periodic substrate renewal or stand turnover could further disrupt continuity of fungal colonization and reduce opportunities for long-term establishment [65]. Such interpretations are consistent with previous findings showing that local site characteristics strongly influence fungal community stability and composition (e.g [62]).

At the same time, the observed differences among stands are unlikely to be attributable to forest continuity alone. Although all sites represent old-growth *Q. robur* stands, they differed in tree admixture, understory composition, herbaceous cover, and soil fertility (see Methods), all of which can influence local inoculum sources, sporulation dynamics, and microclimatic conditions. Fine-scale environmental heterogeneity is well known to generate spatial variation in fungal communities, even over short distances, through microsite-specific reproductive events and localized dispersal [98–102]. Importantly, our study design does not allow us to disentangle the relative contributions of these factors. Consequently, the observed β-diversity patterns likely reflect a combination of site-specific environmental filtering, historical contingencies, and stochastic colonization–extinction dynamics, as predicted by neutral theory [103]. Thus, both deterministic and neutral processes may jointly shape the observed community variation.

Consistent with many studies of airborne fungi (e.g [44, 45, 47–49, 61, 62, 104], Ascomycota and Basidiomycota dominated our dataset. Within Basidiomycota, relative sequence abundance was high relative to OTU richness, indicating that some taxa contributed disproportionately to sequence reads. This pattern is consistent with differences in sporulation intensity during the sampling period and may also reflect variation in propagule production or proximity of sporulating structures to the traps. The prominence of *Agaricomycetes*, *Exobasidiomycetes*, and *Tremellomycetes* accords with the late summer–early autumn sporulation phenology of many basidiomycetes. At the same time, passive spore trapping captures only a fraction of the airborne fungal pool, and taxon-specific differences in spore production and dispersal likely shape the observed patterns, which therefore represent a temporally constrained snapshot of airborne fungal activity.

As hypothesized, saprotrophs dominated the airborne fungal communities (46.5% of classified OTUs), while pathotrophs and symbiotrophs formed smaller yet consistently detectable components of the assemblage. Because saprotrophic fungi release abundant spores while decomposing litter, wood, and other dead organic substrates (e.g [105, 106]), their dominance indicates that ongoing decomposition and nutrient-cycling processes in the surrounding forest are major sources of airborne fungal spores. At the same time, the presence of numerous pathogens and mutualistic fungi suggests that the aeromycobiome also reflects living-host interactions, including plant infections and symbiotic associations, pointing to additional biotic pressures within the forest environment.

The recovery of DNA from red-listed species (*Fistulina hepatica*, *Xylobolus frustulatus*, *Steccherinum bourdotii* and *Fomitopsis rosea*) and invasive pathogens (*Hymenoscyphus fraxineus* and *Ustilago maydis*) even at low relative sequence abundances demonstrates the sensitivity of metabarcoding for detecting rare or invasive taxa, showing its value as a complementary tool to fruitbody- or symptom-based monitoring. In Punios Šilas Strict Nature Reserve, at least 13 red-listed fungi have been reported historically [14, 70], including several oak specialists, yet only three of these were detected by us in Punia oak stand. Such discrepancies may reflect local absences, lack of sporulation during the sampling window, low airborne spore loads (with rare sequences discarded as singletons), or unsuccessful species-level assignment under the ≥ 99% threshold. Although no prior records of invasive fungi exist for the studied stands, the recovery of *H. fraxineus* and *U. maydis* suggests that airborne eDNA can reveal pathogens before visible symptoms occur.

Although detected temporal structuring was modest, detectable variation in airborne fungal community composition reflects the dynamic and episodic nature of fungal sporulation. Many fungal species show short-term or seasonal fluctuations in spore release driven by temperature, humidity, and precipitation [58, 69, 104]. Sporulation and dispersal rates increase under favourable microclimatic conditions, leading to shifts in community composition [59]. Short-lived environmental events - especially humidity fluctuations - interact with species-specific reproductive phenology, generating irregular diel and seasonal sporulation peaks [106]. Although relative humidity frequently emerges as a dominant predictor of aeromycobiome structure, responses are taxon-dependent, and local weather can obscure broad patterns [107]. Collectively, these observations indicate that short-term dynamics in airborne fungal assemblages are governed primarily by the timing and intensity of sporulation across co-occurring taxa rather than by uniform directional change.

Temporal variation in OTU richness and relative sequence abundance likely reflects a combination of biological and physical processes, including changes in local sporulation intensity as well as variation in atmospheric transport and capture efficiency. Wind speed and direction, turbulence, and precipitation can strongly influence particle residence time and deposition, such that similar sporulation levels may yield different sample loads, whereas unfavorable conditions can suppress spore release altogether (e.g [107]). Disentangling biological production from transport effects would require coordinated high-frequency meteorological monitoring and parallel passive and active sampling approaches. Given the compositional nature of metabarcoding data, apparent temporal shifts in relative abundance may therefore arise from both biological changes in sporulation and stochastic variation in atmospheric mixing and deposition. Accordingly, temporal patterns observed here are best interpreted as community-level signals rather than quantitative estimates of spore flux.

Despite these complexities, our data indicate recurrent but moderate temporal turnover in taxonomic composition. Qualitative Sørensen similarity values between consecutive sampling periods (0.58–0.75) point to partial replacement of taxa over time (Table S4; Fig. S6), with the strongest compositional shifts occurring across the August–September transition. Comparable short-term restructuring has been reported in other aerobiological studies (e.g [47, 48, 57, 108]), suggesting that episodic turnover is a common feature of airborne fungal communities.

Changes in relative sequence abundance further support the occurrence of episodic sporulation pulses. Although *Agaricomycetes* and *Dothideomycetes* maintained consistently high taxonomic richness, their proportional representation declined during 7–13 September, when *Exobasidiomycetes*, *Tremellomycetes*, *Eurotiomycetes*, and *Pucciniomycetes* temporarily dominated the assemblage. Such pulses - brief periods during which taxa with narrow or synchronized fruiting windows release large quantities of spores - have been widely documented in fruiting-body phenology and airborne spore studies [109–111]. They result in communities composed of a relatively stable background of common taxa overlaid by transient, high-abundance contributors. Sporulation often responds to fine-scale or lagged environmental cues, such as short-lived humidity spikes, rainfall events, or rapid temperature changes (e.g [52, 53, 58, 59, 106]), and is further modulated by stochastic variation in spore liberation and deposition [59]. Consequently, microclimatic conditions in the days preceding sampling likely exert stronger influence on airborne community structure than the weekly averages available in the present study. Together, these factors reinforce that read-based temporal patterns primarily reflect relative shifts within the sampled community rather than absolute changes in spore production.

Passive spore traps provide an accessible and integrative means of sampling airborne fungi but are subject to methodological constraints, including washout during precipitation, taxon-specific capture efficiencies, and the inability to distinguish viable spores from other fungal propagules or DNA fragments [36, 46, 47, 93]. Consequently, our data reflect the composition of airborne fungal eDNA rather than spores alone. As discussed above, rarefaction analyses indicate incomplete recovery of the rarest taxa, but broadly comparable sampling depth among stands supports robust relative comparisons of community structure. Despite these limitations, passive airborne metabarcoding achieved broad taxonomic coverage and sensitivity to rare and red-listed species, underscoring its utility for fungal biodiversity assessment and monitoring.

Future studies of airborne fungal diversity would benefit from integrating complementary airborne eDNA approaches. Combining passive and active air samplers, quantitative assays (e.g., qPCR or ddPCR), and higher-frequency sampling would improve estimates of spore load, clarify the contribution of transient sporulation pulses, and help disentangle production from atmospheric transport. More systematic use of multi-marker metabarcoding and long-read sequencing may also enhance taxonomic resolution, particularly for understudied fungal groups.

In conclusion, this study provides a robust characterization of the aeromycobiota of old-growth *Q. robur* forests in Northern Europe. Using passive spore traps combined with DNA metabarcoding, we documented high airborne fungal diversity and a substantial core community shared among stands, together with stand-associated differences in species richness and relative sequence abundance. These findings support our first hypothesis, indicating that while many airborne fungal taxa are widespread, local stand characteristics contribute to quantitative differences in community composition.

Temporal variation in airborne fungal communities was also detectable, particularly in species richness and relative sequence abundance. Although ordination analyses showed considerable overlap among sampling periods, statistical testing demonstrated that temporal effects explained a significant but moderate proportion of community variation, providing partial support for our second hypothesis. Functional guild analyses further confirmed that saprotrophs dominated the airborne fungal assemblage, with plant pathogens and symbionts forming smaller yet consistently detectable components, consistent with our third hypothesis.

The agreement of our findings with previous work (e.g. Nordén et al. [92]) highlights the importance of oak forests as reservoirs of fungal biodiversity. Our results demonstrate that passive spore traps-based airborne metabarcoding is an effective tool for detecting both widespread and context-dependent fungal taxa, including rare and conservation-relevant species. Integrating aeromycobiome monitoring into long-term forest surveillance programs could improve assessments of ecosystem condition and fungal responses to environmental change, while future incorporation of quantitative approaches such as qPCR or ddPCR would help link community composition more directly to absolute propagule abundance.

## Supplementary Information

Below is the link to the electronic supplementary material.

Supplementary Material 1

Supplementary Material 2

Supplementary Material 3

Supplementary Material 4

Supplementary Material 5

## Data Availability

All data generated or analysed during this study are included in this published article and its supplementary information file. Sequences of all encountered OTUs are available in the NCBI database (https://www.ncbi.nlm.nih.gov/) under the accession numbers PX688771 to PX690611.
