# Supplementary material for: Metabarcoding Reveals Rich and Diverse Aeromycobiota in Protected Oak Forests
Source: Microb Ecol. 2026 Mar 4;89(1):96. doi: 10.1007/s00248-026-02714-5 (PMC13065563; doi:10.1007/s00248-026-02714-5)
Supplement: Supplementary file 5 — Supplementary Material 5 [file 248_2026_2714_MOESM5_ESM.docx]

**Metabarcoding reveals rich and diverse aeromycobiota in protected oak forests**

Vaidotas Lygis^1^, Adas Marčiulynas^2^, Teodora Plepytė^1^, Sigitas Šulčius^1^, Audrius Menkis^3*^

**Supplementary Figures**


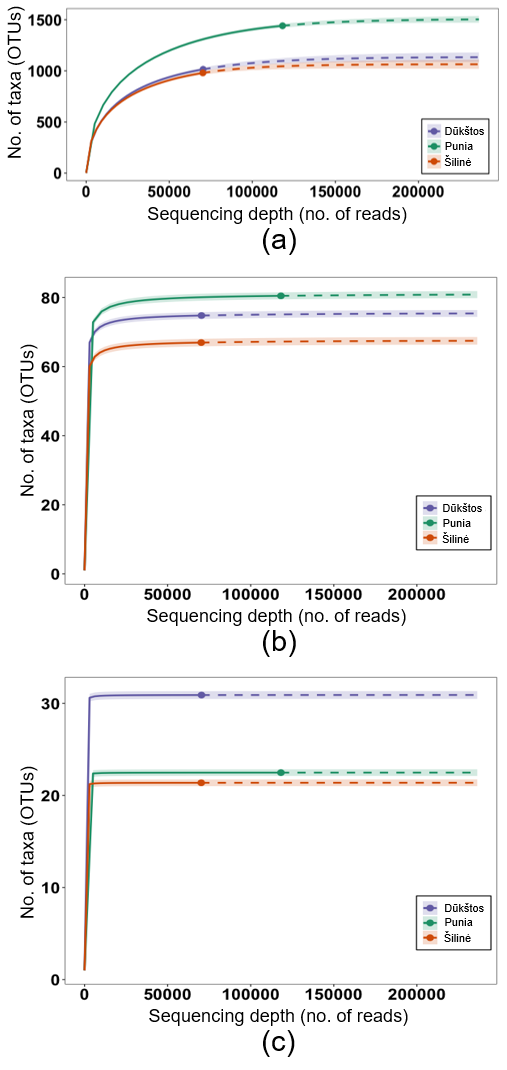


**Fig. S1** Rarefaction and extrapolation curves of airborne fungal alpha diversity for three investigated oak stands (Šilinė, Punia, Dūkštos), showing (a) species richness (q = 0), (b) Shannon diversity (q = 1), and (c) Simpson diversity (q = 2) as functions of sequencing depth. Solid lines indicate interpolation, dashed lines extrapolation, and shaded areas 95% confidence intervals. Points denote observed diversity at maximum sequencing depth. Data are pooled across five sampling periods (August–September 2022)


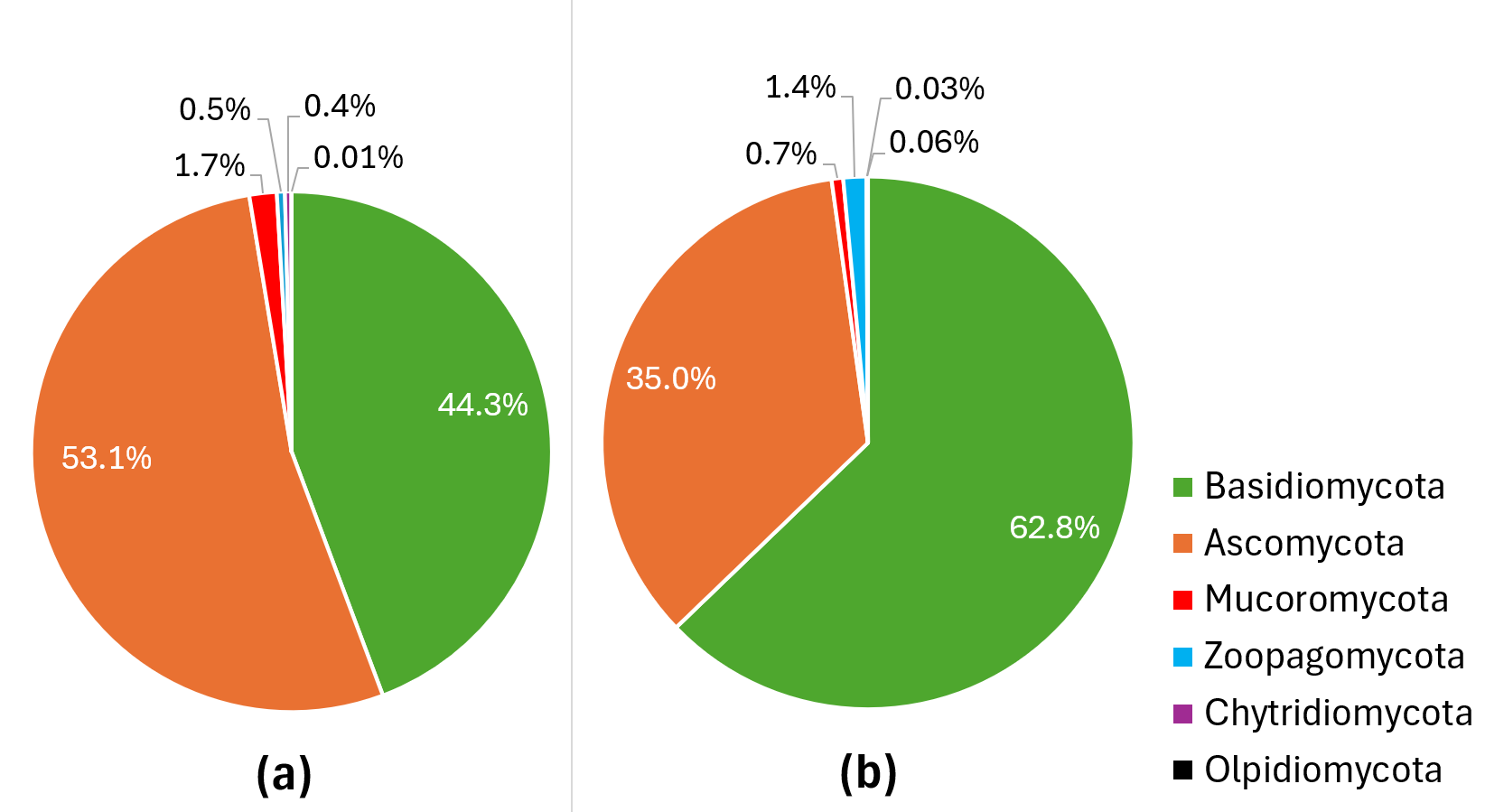


**Fig. S2** Distribution of fungal taxonomic phyla based on the relative abundances of identified taxa (OTUs) (a) and high-quality ITS2 rRNA sequence reads generated by PacBio sequencing (b) in the studied oak stands of Šilinė, Punia and Dūkštos (combined data for all stands)


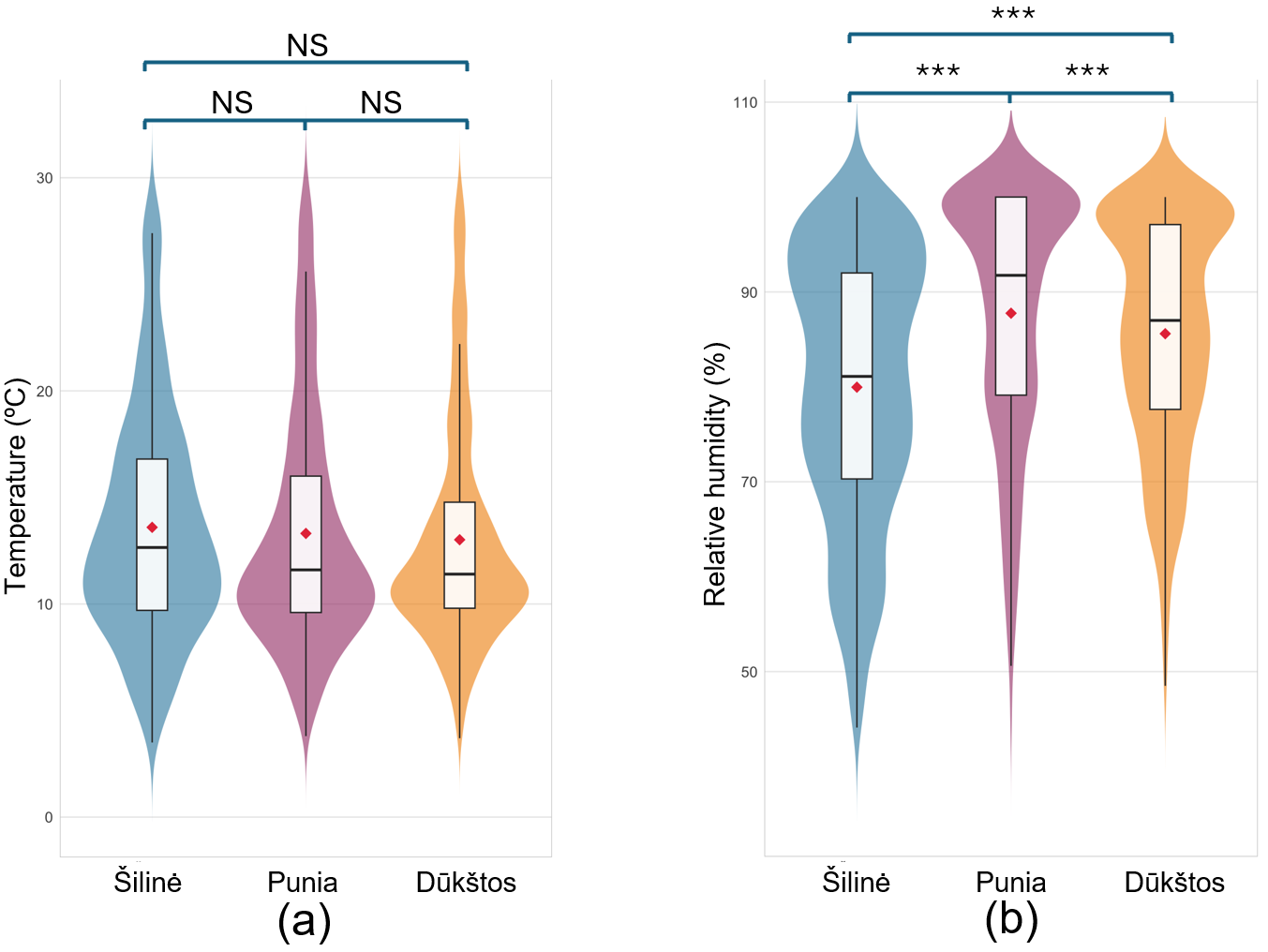
**Fig. S3** Distribution of (a) air temperature (°C) and (b) relative humidity (%) recorded in the investigated oak stands of Šilinė, Punia, and Dūkštos. Violin plots depict the density distribution of measurements, with embedded boxplots indicating medians and interquartile ranges; red diamonds denote mean values. Significance of pairwise comparisons is indicated above plots (****p* < 0.001; NS, not significant)

**Fig. S4** Distribution of fungal classes across the three studied oak stands (Šilinė, Punia, and Dūkštos) based on (a) taxonomic richness expressed as the relative abundance of operational taxonomic units (OTUs), and (b) relative sequence abundance derived from high-quality fungal DNA reads captured by passive spore traps. Data are shown separately for each stand. In panel (a), “Other” comprises fungal classes represented by fewer than 50 OTUs, whereas in panel (b), “Other” includes classes contributing <1.0% of total sequence reads


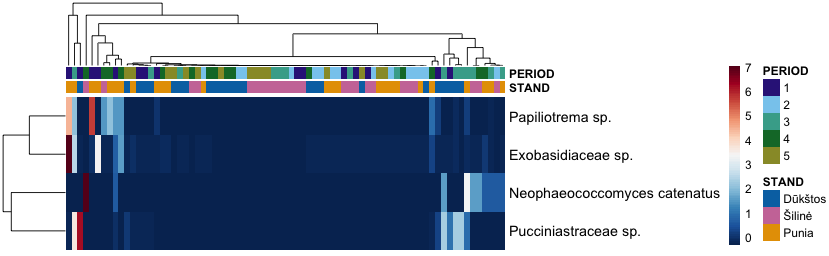


**Fig. S5** Heatmap of relative abundance patterns for fungal taxa identified as differentially abundant among the studied oak stands of Šilinė, Punia and Dūkštos. Rows correspond to taxa and columns to individual samples, with color intensity representing scaled relative sequence abundance. Samples and taxa were hierarchically clustered. Annotation bars indicate sampling period (1–5; see Methods for sampling dates) and oak stand

**Fig. S6** Temporal variation in the distribution of fungal classes based on (a) taxonomic richness (relative abundance of OTUs) and (b) relative abundance of high-quality fungal DNA sequence reads (%) captured by passive spore traps across five weekly sampling periods from August to September 2022. Data are pooled across the three oak stands (Šilinė, Punia, and Dūkštos). In panel (a), “Other” includes fungal classes represented by fewer than 30 OTUs, whereas in panel (b) “Other” comprises classes with relative sequence abundances below 1.0%

**Fig. S7** Distribution of fungal functional guilds in airborne samples collected from the investigated oak stands of Šilinė, Punia, and Dūkštos. (a) Relative taxonomic richness, expressed as the proportion of operational taxonomic units (OTUs) assigned to each functional guild. (b) Relative sequence abundance, expressed as the proportion of high-quality fungal DNA sequence reads assigned to each guild. “Other” includes animal, fungal, and lichen parasites and lichenicolous fungi. Taxa without confident functional assignments were excluded. Many identified fungal taxa exhibit overlapping ecological strategies and were therefore assigned to multiple functional guilds; as a result, the reported proportions should be interpreted with caution
